# Chemical Sensors Based on Molecularly Imprinted Sol-Gel Materials †

**DOI:** 10.3390/ma3042196

**Published:** 2010-03-24

**Authors:** Adnan Mujahid, Peter A. Lieberzeit, Franz L. Dickert

**Affiliations:** Department of Analytical Chemistry and Food Chemistry, Vienna University, Waehringer Strasse 38, A-1090 Vienna, Austria; E-Mails: adnan.mujahid@univie.ac.at (A.M.); peter.lieberzeit@univie.ac.at (P.A.L.)

**Keywords:** sol-gel, molecular recognition, sensors, molecular imprinting

## Abstract

The sol-gel technique is earning the worldwide attention of researchers in the field of material science, due to its versatility in synthesizing inorganic ceramic materials at mild conditions. High purity, homogeneity, controlled porosity, stable temperature and nanoscale structuring are the most remarkable features offered by this method for generating highly sensitive and selective matrices to incorporate analyte molecules. The crafting of sol-gel sensors through molecular imprinting has put great influence on the development of innovative chemical sensors, which can be seen from the growing number of publications in this field. The review provides a brief overview of sol-gel sensor applications, and discusses the contribution of molecular imprinting in exploring the new world of sensors.

## 1. Introduction

**Figure 1:**
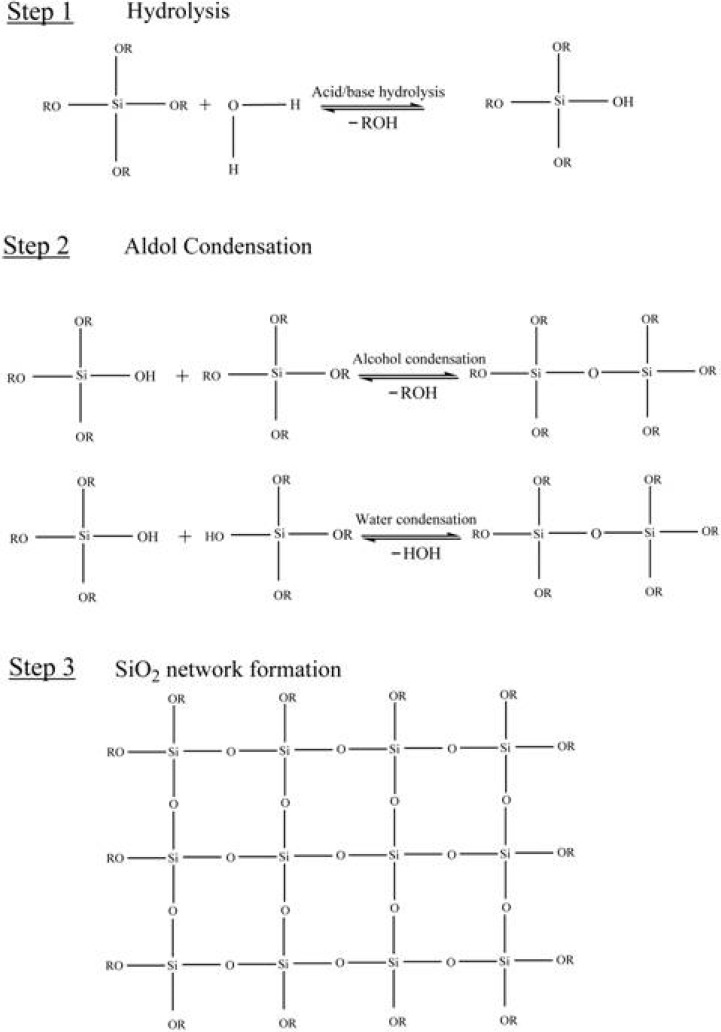
Step-wise description of the sol-gel process.

The sol-gel technique is one of the most promising tools in material science. The versatility of this method allows us to design desired materials at low temperatures, alternatively to conventional methods for manufacturing glass and other products. The synthetic route provided by this system is the most feasible one for designing materials possessing unique properties. Generally, it is a process concerning transition of a system from liquid ‘sol’ (the colloidal suspension of particles) into solid ‘gel’. There are three steps for sol-gel processing; the first method is gelation of colloidal particles, the second is the hydrolysis and poly condensation reactions of alkoxide precursor followed by hypercritical drying, while the third method is similar to the second one: the only difference is in the drying process, which takes place at ambient temperatures. Metal alkoxides are very famous precursors; as soon as they are exposed to water they are hydrolyzed. The partially hydrolyzed alkoxide molecules react with each other or with the un-hydrolyzed ones to undergo a condensation reaction and form a cross-linked matrix liberating water or alcohol. The schematic representation of the sol-gel process is illustrated in Figure 1.

When the reaction is in progress, the viscosity of the matrix increases gradually depending upon the degree of cross linking, and ultimately turns into an interconnected, homogenous and rigid material, which after aging, is dried at room temperature to obtain final product. The two-step sol-gel reactions largely depend on several parameters such as solution pH, type and concentration of the catalyst used for hydrolysis, reaction temperature, heating time, nature of the (R) group attached and the solvent used for this process. It has been noticed that these different variables have a large influence on the fundamental characteristics of sol-gel materials such as homogeneity, porosity, refractive index, surface area and mechanical and thermal properties. The physical and chemical principles involved in sol-gel processing and its applications have been excellently addressed in reviews [1,2,3,4]. The most comprehensive work on this topic had been presented by C. Jeffrey Brinker [5] in his famous book *‘SOL-GEL SCIENCE’*.

The materials formed under sol-gel technology have found numerous applications in various fields, such as the glass industry, ceramics, and thin films, different biological and chemical sensors. There are some inherent features of sol-gel materials that make them a promising tool for sensing applications. Sensitive material can be generated by adding some recognition element in the sol-gel matrix during synthesis, which does not interact chemically with the surroundings. This recognition or template molecule is engulfed within the sol-gel matrix. The process of engulfing the template to yield functionalized sol-gel materials is tailored by doping or grafting procedures. Doping is a physical phenomenon in which template molecules are entrapped by polymer chains while grafting involves the formation of covalent bonds. The doping technique is very simple and virtually applicable to all systems in comparison to the grafting method. The only problem associated with doping is the leaching of template, which is a major factor in the control of pore size. The major advantage of sol-gel materials is in formation of thin films of extremely small layer heights *i.e.*, in the nanometer range. These thin layers are produced by spin coating or drop coating methods. The speed of the spin coater helps us to control the thickness of the films. The potential application of these thin films is to develop optical sensors as they are transparent in the visible region [6,7]. These sensors are employed for detection of metal ions [8,9,10], monitoring of pH [11,12,13,14,15,16,17] and in several other fields. The sol-gel films can also be coupled with optical fibers to develop wave sensors [2].

Molecular imprinting [18] is a smart way to design artificial antibodies that are used to generate recognition characteristics within the polymer matrix. In this method, a template is added in the polymer matrix, the polymer chains self-organize around the template molecules, which can be detached by heating or washing methods without disturbing the geometry of the polymer. The removal of the template from the polymer matrix leaves behind very fine adaptive cavities that are capable of reversible re-inclusion. Introducing the imprinting effects to sol-gel science, influential sensing materials can be designed that can be employed for different biological sensing and environmental monitoring. Molecular imprinting has the ability to create optimized template sensitive cavities for analyte re-inclusion without interacting chemically with the polymer system. On the other hand, inorganic ceramic sol-gel materials of desired properties can be produced that can face up to the harsh temperature environments while keeping their tailored features alive. The combination of these two strategies leads to the development of highly sensitive and robust materials that possess synergic effects. Although the application of molecular imprinting to sol-gel materials for producing sensors is in infancy, it is promising for advances towards modern sensing systems.

In this review article, we present the research in sol-gel materials considering their applications in the field of chemical sensors. The investigations made are in progress to synthesize innovative imprinted sol-gel sensor materials. This work briefly explains the unique ways in which imprinted sol-gel surfaces can be employed for sensor applications.

## 2. Sol-Gel Technology

Some 150 years ago, Ebelman [19] and Graham’s [20] studies on silica gels gave birth to a new dimension in material chemistry *i.e.*, inorganic sol-gel processing. The initial studies were carried out on tetra ethyl oxy silane Si (OC_2_H_5_)_4_ to synthesize SiO_2_ networks under acid catalyzed reactions. Since then, the sol-gel science has become the most pronounced strategy in material design. The reaction is accomplished by hydrolysis of metal alkoxide M(OR)_n_ precursors and poly condensation steps in liquid medium e.g., alcohols or typical low molecular weight solvents. Once the hydrolysis reaction initiates, the condensation propagates, leading to the formation of sol-gel networks and some other by-products like water or alcohols, which can be easily removed by the drying processes. The two reactions go on side-by-side, and in this way the viscosity of the system gradually increases. At this point, the gels interconnect with each other and develop a strong network. The reaction can be stopped or prolonged during the course of gel formation to obtain final products of required features.

Early investigations were focused on silicates only; later on, the concept of sol-gel was extended to titanium, zirconium or metal alkoxide. These precursors react with water more vigorously as compared to the silicates. It has been noticed that complete drying of the solvents produced during the reaction leads to the formation of less porous materials as compared to hydrated ones.

The growing interest in sol-gel science is due to the straightforwardness in tailoring the porosity in these materials, particularly in achieving molecular recognition in different systems such as adsorption or absorption, stationary phases in liquid or gas chromatography and chemical sensing applications. The production of sol-gel materials provides numerous advantages over the other conventional synthetic schemes. One of the major advantages is that these systems allow the manufacturing at low temperatures, which enables us to incorporate the template molecules as recognition elements to generate functionalized matrices, where others lack this ability. These materials are synthesized from solutions that help us to generate thin films and bulk products of desired shape. In this process, the chemical compositions of the starting material controls porosity by carefully monitoring the reaction parameters. The thin sol-gel films can easily be immobilized on glass and other silicon substrates through covalent linkage, which is an additional feature of these materials. The end product material can be configured in different forms like powder or dispersion forms, thin films, fibers and monoliths, *etc.* One of the most exciting achievements of this technique is that a hybrid material of organic and inorganic substances can be synthesized. These hybrid materials are named *ormosils* [21,22] (originally modified silicates), which contain both flavors of organic and in-organic properties. These materials can be modified according to their well suited applications in respective fields by varying the composition of precursors, catalyst and other reaction conditions.

## 3. Sol-Gel Based Chemical Sensors

Extensive study has been made on the sol-gel technique for the development of different chemical sensors during the last few years. The major use of this technology is in the field of optical sensors, however electrochemical and biosensors are other areas of interest that cannot be ignored.

### 3.1. Optical Sensors for pH Determination

Optical sensors based on sol-gel thin films offer significant advantages over other sensing systems, because they are based on thin films produced by sol-gel methods. When light interacts with the thin films, the optical properties change, which is normally induced by adding some optical sensitive molecule or by varying some external physiochemical parameters such as temperature, pressure or some radiations. Changes in behavior of the thin films are monitored by processing optical signals. One of the most reported optical sensors based on sol-gel thin films are pH sensors. These sensors are highly sensitive, in-expansive and show reversible and quick responses. They are produced by incorporating a pH indicator or pH sensitive dyes into the sol-gel matrix. Most optical sensors used for pH determination are based on fluorescence or absorbance measurements. The combination of optical fibers with sensitive sol-gel thin films can lead to the development of specific pH sensors, which have broad range and shorter response time. This idea has been practically implemented by Suah *et al.* [23] to design a pH sensor based on optical fiber. They used bromo phenol blue as a doping indicator, which showed a much improved pH range *i.e.*, 2.0–12 with 15–150 second response time( previously 3.0–5.0 for the same pH indicator in the same sol-gel matrix). McCulloch *et al.* [24] made a step forward to develop miniaturized pH sensors based on optical fibers in which they coated pH sensitive sol-gel film on an optical fiber of sub-micrometer dimension.

In order to obtain an accurate, precise, highly sensitive and broad range pH sensor, mixture of multiple pH indicators [25,26,27,28] are introduced into the sol-gel matrix. These sensors are not only used for typical pH measurements but are also employed in different industrial applications for degree of acidity measurements. They offer reliable, sensitive and cost-effective detection systems as compared to other conventional measurements.

### 3.2. Sensors for Ionic Compounds

**Figure 2:**
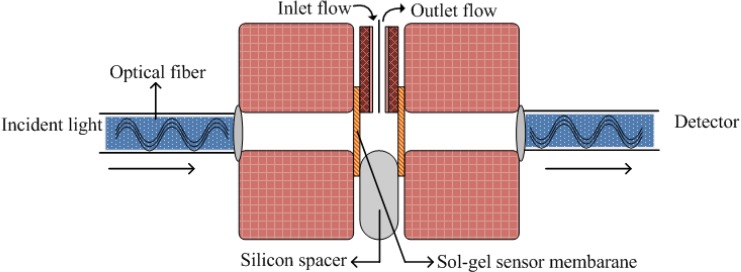
A schematic representation of the Cu^+2^ ion sensor sep-up (adapted from Ref. [33]).

Thin silane films have found numerous applications for optical sensing of several ionic species such as different metal ions [29,30,31,32,33,34,35,36]. Sol-gel materials help us detect metal ions in two ways: firstly, the silane groups offer sufficient sensing sites to metal ions as they possess a large surface area, and secondly, due to the presence of entrapped recognition molecules. These features provide extra-ordinary sensitivity for metal ion detection. The entrapped ligand in the sol-gel matrix possesses sufficient freedom to reorient for complex formation with target metal ions. Recently, an optical sensor was developed where 4-(2-pyridylazo) resorcinol [PAR] is physically entrapped in thin sol-gel films for determination of Cu^+2^ ions [33] in urine samples. This entrapment in thin films is based on a base-catalyzed strategy. The proposed optical sensor for Cu (II) shows high sensitivity, selectivity, stability and short response time. The schematic diagram for this sensor is shown in Figure 2. The developed sol-gel based optical sensor for Cu^+2^ ions is very suitable for diagnostic purposes. There is no substantial difference in the results obtained by the sol-gel optical sensor and from inductively coupled plasma mass spectrometry (ICP-MS) at a confidence level of 95%.

Sol-gel films are not used for cationic species, but were also proposed for anionic species at a relatively smaller scale. Dunuwila *et al.* [37] develop CN^-1^ sensors by entrapping iron (III) porphyrin in thin sol-gel films of titanium carboxylate. Due to the irreversible reaction of CN^-1^ with entrapped dye, test strips were designed for single-use only. The sol-gel anion sensor shows high sensitivity and a lower detection limit. The best example is a sensor for chromate ion [10], which possesses a detection limit of 1 part per billion (ppb). Another common example is halogen anion (Cl^−^, Br^−^, I^−^) sensor [38], which was developed by Jiwan and Soumillion.

### 3.3. Gas Phase Sensing

**Figure 3:**
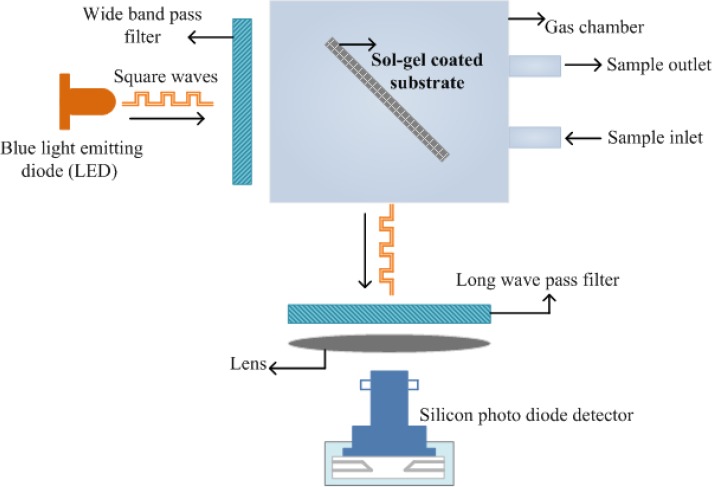
Representation of experimental set-up used for optical sensing of oxygen in both gas and aqueous phase (adapted from Ref. [44]).

Several sol-gel based optical sensors have also been proposed for detection of different gases such as oxygen, carbon dioxide, ammonia and many others. The growing demand in gas phase detection [39,40,41,42] led to the development of modern sensor systems based on thin sol-gel films, which are considered more useful than the usual amperometric methods. The importance of CO_2_ cannot be denied in environmental, industrial and several biological processes. Malins and MacCraith [43] designed CO_2_ gas sensors by immobilizing fluorescent dye pyranine in a hydrophobic sol–gel ormosils derived glass using phase transfer reagent. McDonagh *et al.* [44] developed a sol-gel based optical sensor for detecting oxygen in gaseous and liquid phase. The determination of oxygen in liquid or gas phase has significant importance due to industrial, biological and environmental applications. They employed porous sol-gel films based on a ruthenium complex for oxygen sensing. The principle of sensing is based on fluorescence quenching of ruthenium. A schematic representation of designed oxygen sensor in both liquid and gaseous phase is shown in Figure 3.

The detection of nitrogen dioxide in the low concentration range has found substantial importance in different industrial processes, environmental monitoring, biosensors and various other fields. Worsfold *et al.* [45] designed NO_2_ gas sensors based on sol-gel entrapped prophyrins that were deposited as thin sensitive film on glass substrates. Grant *et al.* [46] employed the sol-gel based fiber optical sensors for monitoring nitrogen dioxide considering two different strategies. In the first case, Saltzman reagent was used, which showed good sensitive but irreversible signal, which is not suitable for continuous monitoring. The second method was based on ruthenium complexes, which have shown promising results with rapid response times. Sol-gel films are highly efficient for sensitive detection at extremely low concentration levels. Mechery and Singh [47] developed an optical fiber based sensor for very sensitive and selective detection of nitrogen dioxide in air samples. They attained the ppb level detection limit using the Saltzman reagent entrapped in the sol-gel matrix.

### 3.4. Biological Sensors

The major achievement in sol-gel sensor technology is the successful induction of several biological recognition elements such as enzymes, bacteria, proteins and even whole cells. The exciting feature of this strategy is that the entrapped biological recognition element does not lose its structural functionality and retains original characteristics. Thin and transparent sol-gel films provide suitable platforms for the detection of several biological species with enhanced sensitivity and selectivity. The conventional sol-gel route is not feasible for the encapsulation of different biological agents, as the low pH value and presence of alcohols denature different proteins, which is a serious issue concerning the functional and structural integrity of that particular specie. The problem can be solved by modifying the synthetic route. For example, a suitable buffer solution is used after an acid or base catalyzed hydrolysis reaction and the typical quantity of alcohols is reduced. This allows us the successful encapsulation of biological species in thin sol-gel films without losing their character, which ensures the development of a promising biosensor. The first application of the sol-gel biosensor [48,49] was based on interactions between hemoglobin, myoglobin for CO and NO.

**Figure 4:**
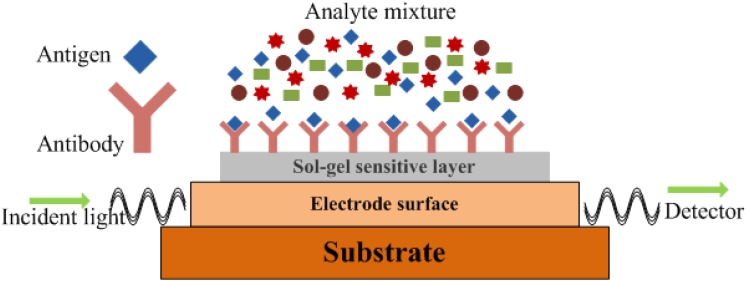
A typical set-up of an optical immune sensor (adapted from Ref. [3]).

One of the most attractive examples of this field is immune sensors, in which a biological recognition molecule is attached to a sensing surface via the sol-gel route. A simple optical immune sensor can be represented by a schematic diagram as shown in Figure 4. The optical signals coming from the sensitive surface are based on refractive index (RI), fluorescence, luminescence, absorption, emission or light polarization. These immune sensors have gained considerable interest due to their high sensitivity and selectivity in bio analytic measurements, especially for the detection of different bacteria, viruses and endotoxins. The importance of these pathogenic species is not only in clinical analyses, but also in different industrial, environmental and military applications. The flexibility of the sol-gel technique allows the crafting of several optical biosensors [50,51,52,53,54,55,56] of desired need.

## 4. Molecular Imprinted Sol-Gel Materials as an Innovative Sensing Tool

**Figure 5:**
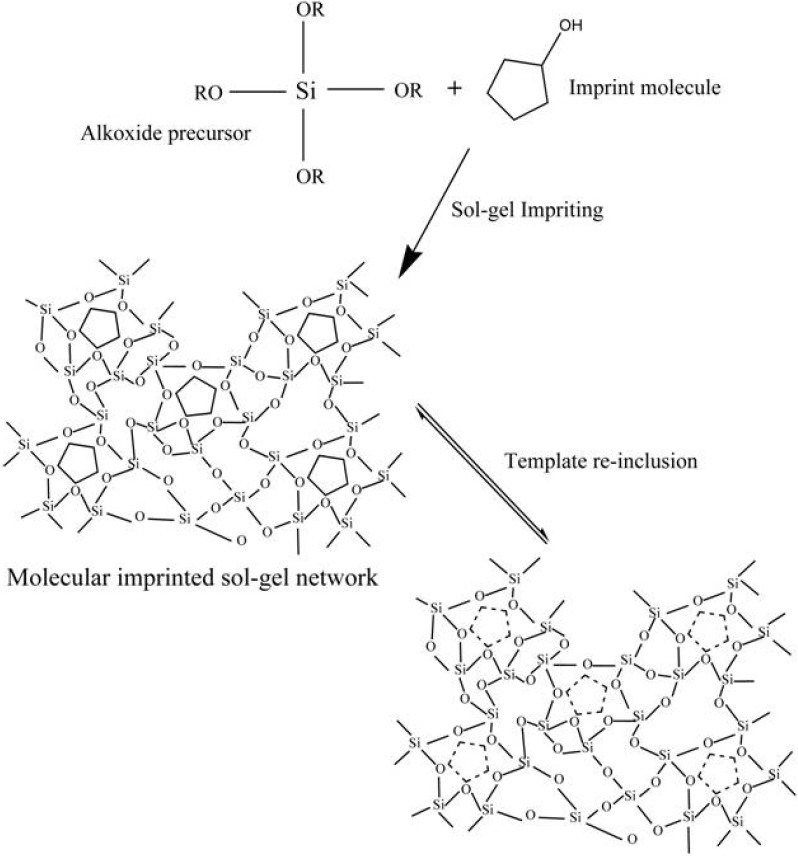
Description of molecular imprinted sol-gel processing (adapted from Ref. [70]).

Molecular recognition concept is associated with the lock and key mechanism, which was first reported by Pauling [57] to explain the recognition theory. This idea is successfully translated by the molecular imprinting technique, which was first introduced by Kiefer *et al.* [58] and Wulff [59], independently in synthetic organic polymer systems. Since then, this scheme has found numerous applications in separation processes, immunoassays, biosensor recognition systems and several other chemical sensors. Basically, molecular imprinting is of two types: one is based on covalent binding reported by Wulff [60] and the other is non-covalent interactions Mosbach [61,62], where both techniques possess their own advantages and disadvantages. A typical example of molecular imprinting in sol-gel system is presented by the following reaction scheme (Figure 5). It is obvious from the diagram that the imprinted polymer retains the shape and size of template after its removal. Thus, the molecular imprinted polymer (MIP) acts as sensing material for this particular analyte.

On the other hand, sol-gel, as earlier mentioned, is a convenient method to design stable and robust materials at ambient conditions. The high degree of cross-linking in these material such as (SiO_2_)_n_ network is a very useful property that is employed to synthesize imprinted materials. The high thermal stability of sol-gel materials helps us in the removal of template molecules by allowing heating methods at higher temperatures without losing the characteristics of developed recognition cavities [63]. The porosity of these materials provides high surface area (200–2000 m^2^/g) for analyte interactions, which play important roles in the sensitivity tuning of MIPs. Additionally, the desired selectivity in sol-gel MIPs can be achieved by selecting a suitable precursor. Introducing an in-organic template into an organic polymer is also a significant success of these materials. The control in reaction conditions and parameters leads to the formation of thin films having the required degree of porosity, thickness and surface area that ensure the production of selective and sensitive MIPs. A typical example of a molecularly imprinted sol-gel material can be best understood by the model diagram presented in Figure 5.

**Figure 6:**
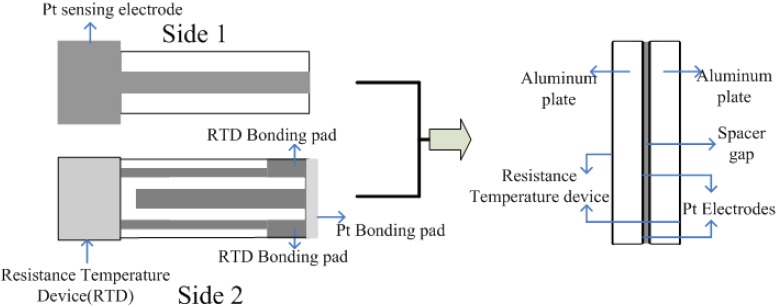
Oil conditioning sensor based on conductometric measurements (adapted from Ref. [64]).

The analysis of degraded engine oil is a very difficult assignment, as automotive engine oil comprises of a number of ingredients that overall tend to improve the performance. During the course of time, the engine oil undergoes several degradation phases where long chain hydrocarbons are cracked down. Although the exact life time of engine oil is not known, it is quite necessary to drain the degraded oil to avoid any complications in engine operations. Therefore, continuous monitoring is needed for this purpose. The classical sensor systems used for monitoring the quality of degraded oil provides very little information about certain physical or chemical parameters only. A typical oil condition sensor [64] based on conductometric measurement is shown in Figure 6. Some of them investigate the electrochemical behavior of the degraded oil such as voltammetric and conductometric parameters while other involves the acid base titration methods. These sensor systems are silent about the molecular recognition, which is key point in sensor development.

Sol-gel material imprinted with certain recognition molecules provides answers to all the questions related to the molecular recognition of degraded engine oil. Focusing on automotive engine oil degradation analysis [65], molecular imprinted sol-gel layers have found outstanding sensor applications employing quartz crystal microbalance (QCM) for mass sensitive measurements. Ceramic sol-gel [66] layers having functional tertiary amino groups based on polymerization of silicon alkoxide have been used as a sensing surface. This surface ensures the optimized interactions with all sorts of acidic products coming out from the degraded engine oil. Stability at elevated temperatures and porosity of these ceramic sol-gel layers impart unique features for sensing applications. Capric acid (Decanoic acid) is used as a recognition molecule in imprinting procedures due to its suitable length and solubility for the detection of a representative class of carboxylic acids. The presences of tertiary amino groups also contributes some hydrogen bonding in addition to van der Waals interactions. The synthesis of molecular imprinted silane sol-gel layers is carried out at ambient conditions, which is advantageous over conventional organic polymer preparations. The template molecule *i.e.*, capric acid, can be removed from the matrix by washing the layers with toluene or by some heating methods. Infra red (IR) studies before and after removal of capric acid show a significant difference in the absorbance intensities. The removal of the template produces very sophisticated cavities for selective incorporation of analyte molecules. These sensitive layers are deposited on a mass sensitive transducer surface such as QCM. Results obtained from these devices shows a considerable difference between fresh oil and degraded automotive engine oil. These results enable us to recognize the degradation point in engine oil and suggest its time of replacement. The imprinted silane sol-gel layers [67] are competent for oil degradation analysis below 120 °C. They are not effective when the temperature of the working environment is about 150 °C as the silane layers oxidize at such a high temperature and lose imprinting character and stability.

**Figure 7:**
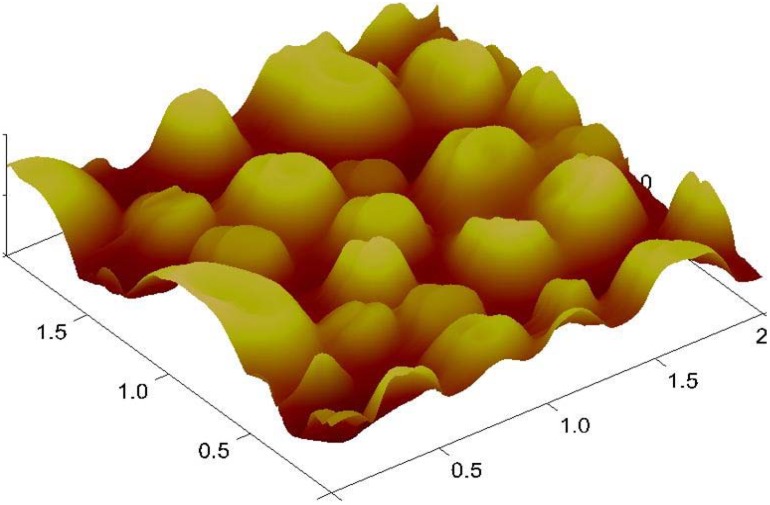
AFM image of the molecularly imprinted sol-gel Titania layer.

The problem is overcome by introducing titanium alkoxide as a precursor. The preference of choosing titanium over silane is due to its high temperature resistive nature, while the rest of the strategy is the same as for oil degradation analysis. Imprinted titanium sol-gel layers [68] are highly stable at elevated temperatures and do not undergo oxidation when exposed to degraded engine oil, particularly in harsh environments. The cavities generated by imprinting procedures are robust and do not immediately collapse when expose to high temperatures. The AFM image of the titanium sol-gel layer is shown in Figure 7 at a scale of 2 µm.

**Figure 8:**
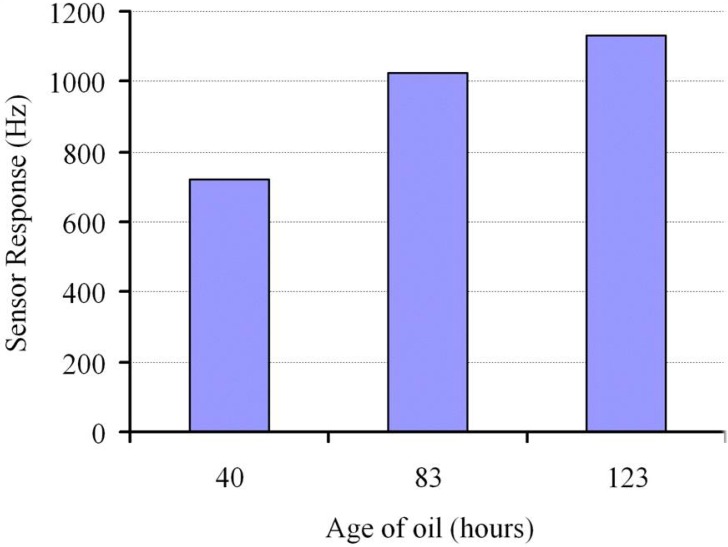
Sensor response for different aged engine oil samples. A clear distinction can be made between different aged samples by using imprinted sol-gel Titania layer as sensor coating.

This image shows an excellent imprinting pattern of the titanium sol-gel layer. These cavities selectively incorporate the acidic products from degraded engine oil. Mass sensitive measurements performed with different used engine oil samples exhibit appreciable sensor responses, as shown in Figure 8. It is obvious that different aged oil samples are clearly distinguished from their respective sensor signals. Titanium sol-gel layers are superior over silane layers in two regards; first in sensitivity and second in stability at extreme temperatures. The lower electronegativity value of titanium compared with silicon makes Ti-O- bonds more polar in comparison to Si-O-, thus making titania sol-gel layers more sensitive for analyte incorporation. The sensitivity is also enhanced due to the basic nature of titania that favors the interactions with acidic products from used engine oil. Moreover, the analyte mass bonded to sol-gel surface is rigid and the viscosity effects do not interact with the QCM device. The contribution of molecular imprinted sol-gel sensors is not limited for degraded engine oil sensing but it has broad range sensor applications [69].

A number of reviews [70,71,72] on molecularly imprinted sol-gel materials have been presented concerning their sensor applications in recent years. Molecularly imprinted sol-gel polymers can be used effectively for designing highly sensitive materials for explosives detection. Walker *et al.* [73] developed an innovative sensor for 2,4,6-trinitrotoulene (TNT) detection in gaseous phase, exploiting integrated optical wave guide attenuated total reflection spectroscopy. They selected TNT as a model compound and adopted a hybrid approach for the imprinting process using silanes. The authors prepared different imprinted films with different proportions of monomers for an optimal polymer recipe to lower detection limits. The designed polymer films show substantial sensitivity *i.e.*, in ppb, and exhibited a linear signal in the concentration range of 4−10 ppb. The selectivity of the TNT sensor has also been examined by exposing 2,4-dinitrotoulene (DNT), 3-nitrotoulene and toluene under the same protocol. It was found that imprinted sol-gel films show a minor effect for these analogous compounds. The high sensitivity and selective nature of MIP sol-gel materials along with integrated optical wave guides make this detection system greatly useful for explosives detection, which has relatively low cost.

Environmental protection is one of the greatest challenges faced by scientists in the rapidly developing world. Chlorinated insecticides such as 1,1 bis[4-chlorophenyl]-2,2,2-trichloroethane (DDT) have been widely used in different industries, agriculture and other household purposes, which has potential hazards for human beings. Edmiston *et al.* [74] designed sol-gel derived materials for sensitive and selective detection of DDT following a sacrificial spacer imprinting approach. They introduced **7-**nitrobenz-2-oxa-1,3-diazole, (NBD) as a fluorescent reporter to monitor the binding of DDT with the MIP interface. The developed sensor surface shows suitable selectivity and high sensitivity e.g., DDT detection limit of 50 parts per trillion (ppt) in aqueous solution. Molecularly imprinted sol-gel films have also been used in developing modern sensors for other insecticides such as paratheion. In [75], the binding of paratheion with sol-gel imprinted polymer films in liquid and gas phase was monitored using cyclic voltammetry (CV) and QCM, respectively. The imprinted sol-gel films were characterized for binding by using gas chromatography flame photometric detector (GC-FPD). The imprinted cavities show high selectivity over other closely related organophosphates compounds and quite reasonable sensitivity in liquid phase analysis by CV. Despite the fact that the gas phase measurement for paratheion sensing is less significant due to increased unspecific binding, the overall approach for liquid and gas phase analysis is attractive one.

The group of Edmiston [76] extended the hybrid approach of molecular imprinting to design fluorescence based chemical sensors for flourene detection. Flourene is a polycyclic aromatic hydrocarbon (PAH) and found in very low concentrations, which are difficult to detect. Molecularly imprinted bis (trimethoxysilylethyl) benzene (BTEB) sol-gel material along with fluorescence spectroscopy provides a highly sensitive platform *i.e.*, in ppb range, for flourene sensing. The selectivity of the imprinted layer was examined by determining the relative signal of flourene analogous compounds such as fluorene-2-carboxaldehyde, anthracene, fluoranthene, naphthalene. Moreover, the control sol-gel film did not show any significant fluorescence intensity for flourene and other test compounds. The authors suggested that the reversibility of the sensor signal can be improved by modifying the binding site. The sensitivity and the detection limit can be further enhanced by favoring the polar interactions of the template with the sol-gel surface.

Despite the fact that conventional sol-gel chemistry has introduced many sensor materials, molecular imprinting has given a new spin to this field in developing more sensitive and selective matrices with remarkable recognition properties. One such example was reported by A. Fern- andez-Gonzalez *et al.* [77], where the performance of molecular imprinted sol-gel films compared with monolithic sol-gel for selective detection of nafcillin *i.e.,* an antibiotic used in treatment of infections caused by gram positive bacteria. The specific binding sites of imprinted sol-gel films were characterized by room temperature phosphorescence (RTP) assay. Different compositions of imprinted sol-gel films were investigated to design optimal binding cavities for nafcillin recognition. There was considerable difference between RTP intensities of imprinted sol-gel monolithic and the corresponding control. The detection mechanism based on imprinted sol-gel RTP assay is very useful for screening of nafcillin in various biological samples. This strategy was further explored to examine the nafcillin amounts in commercial samples *i.e.*, milk based products. In these studies [78], the effect of sample matrix on the imprinted sol-gel sensitive film and on the transducer processing was examined. This proves to be very useful to extend the nafcillin analysis on real samples from commercial products. The lowest detection limit achieved for skimmed and aqueous milk was in the range of 10^-5^ and 10^-6^ mol/L, respectively, with relative standard deviation of less than 5%. The statistical studies show that the recognition of nafcillin is not disturbed by different sample matrices, indicating ruggedness of the analytical model for antibiotic sensing.

Tao *et al.* [79] proposed an innovative sensor strategy for selective and quantitative determination of proteins. They developed protein imprinted xerogels with integrated emission sites (PIXIES) for protein recognition, which showed excellent results. The authors successfully introduced luminescent reporter molecule in PIXIES to monitor the imprinted polymer protein binding, and thus transducing this event into an appropriate signal. The limit of detection ( achieved by this method was about 2 pg/mL that is appreciable while dealing with low concentrations. The cross sensitivity was also examined by exposing ovalbumin imprinted PIXIES to human serum albumin (HSA), and it was found that the sensor signal for target specie is much higher than the other. The synthetic approach for bio recognition is advantageous over natural antibodies as they exhibit almost reversible binding interactions and are suitable for long term analysis. As in this case, the reported reversibility was about 95% after performing more than 25 analyses. The obtained analytical data from PIXIES was comparable to the enzyme linked immune sorbent assay (ELISA) method in terms of detection limit, response time and selectivity factor. In all these aspects, PIXIES materials showed better results in comparison to a relatively expansive method *i.e.*, ELISA. The PIXIES analytical model for protein detection possesses outstanding features as mentioned, which is due to the synergic effect of molecular imprinting and sol-gel method. A piezoelectric sensor coated with molecularly imprinted sol-gel layer was reported for histidine by Zhang *et al.* [80]. The imprint molecule *i.e.*, histidine, was imprinted in silane sol-gel material without any pre-treatment. Histidine imprinted sol-gel film was coated on AT-cut QCM and characterized in air and buffer solution by impedance measurements. Scanning electron microscopy (SEM) was also used to study the swelling of imprinted films prepared by different compositions of precursors in air and buffer solution. The performance of the sensor was tested by micro gravimetric analysis and by electrochemical detection through cyclic voltammetry measurements. The results from these methods exhibit linear sensor response for L-histidine in the range of 10^-8^ M–10^-4^ M with a detection limit of 2.5 × 10^-8^ M. The pH effect was also noted for L-histidine detection in order to obtain an optimized sensor signal. It was found that the frequency shift was maximum at pH 7.4, which is near to the isoelectric point of L-histidine *i.e.*, 7.59. The designed sensor material demonstrates good stereo selectivity with an appreciable recovery for template molecules. A similar strategy was proposed for selective detection of cytidine using piezoelectric quartz crystal gold electrode by the same author [81]. In this case, the quartz surface of the gold electrode was modified by thiols and then gold nano particles were covalently bonded with thiols. After this treatment, the modified electrode surface was exposed to monomer solution *i.e.*, 3 amino propyl trimethoxy silane (APTMS). Cytidine was introduced directly on this surface to conduct imprinting procedures. This synthetic scheme is somewhat different from other sol-gel imprinting methods, as in this case all the synthesis is carried out on the transducer surface, which is similar to layer by layer (LBL) assembling approach.

Selective detection of hormones by organically modified silica is another important example of molecular imprinted sol-gel materials. In [82], acetic anhydride was used as a solvent for sol-gel preparations of silica to better dissolve hydrophobic template *i.e.*, cholesterol. The cholesterol imprinted silica surface exhibited excellent porosity, which selectively adsorbs steroid hormones like progesterone from organic solutions. The adsorption of different steroid hormones were tested on cholesterol imprinted silica adsorbent and their adsorption coefficient was determined. It was noted that the Chol-H silica demonstrates maximum percentage adsorption for progesterone in comparison to steroid hormones and analogous substances. The development of synthetically imprinted silica materials for selective hormone detection is a significant achievement for developing sensors in bio-analytical field.

Recently, another example of cholesterol imprinted microporous silica was reported [83], where the authors attached cholesterol with a function monomer and then proceeded towards the sol-gel process. This led to an improved imprinting factor in silica material for cholesterol. The imprinted sol-gel silica catalyzed with HCl preferably at low pH values showed better imprinting effects as compared to ammonium hydroxide catalyzed polymer. The cholesterol imprinted sol-gel silica exhibited much better adsorption for cholesterol than vitamin D3 and other steroid hormones.

**Figure 9:**
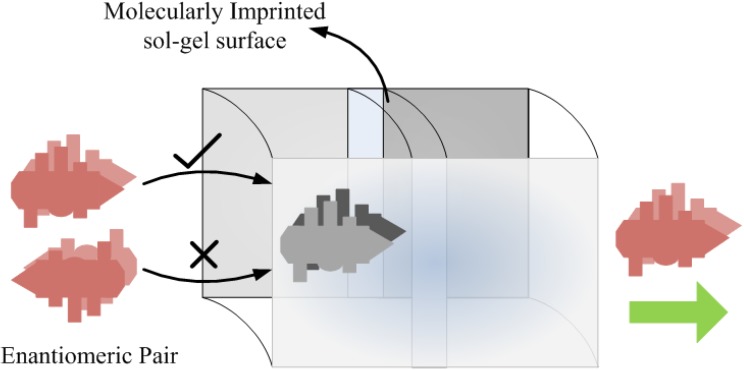
Scheme of an enantiomeric sensor based on molecularly imprinted sol-gel technique (adapted from Ref. [78]).

Molecular imprinted sol-gel technique has also been utilized in the pharmaceutical industry for separation purposes [84]. Lisinopril dehydrate, which is a pharmaceutical active in various drug formulations, has successfully been imprinted in acrylic sol-gel polymer and tested on high performance liquid chromatography (HPLC). The imprinted sol-gel material displayed enhanced selectivity for lisinopril extraction in related substances and other non-active drug ingredients. The designed material showed reproducible results over an eight month time, which indicates the robust nature of the synthetic polymers. Leung *et al.* [85] developed an innovative sensor matrix based on molecularly imprinted sol-gel luminescent sensing material for a very sensitive detection of hazardous herbicide. The detection of non-fluorescent herbicide *i.e.*, 2,4-Dichlorophenoxyacetic acid, has been made possible by using photo induced electron transfer (PET) mechanism. The application of PET for the sensing purpose in synthetic polymers *i.e.*, MIP sol-gel materials, seems attractive to impart fluorescence properties in non- fluorescence compounds, which is quite useful for signal transduction. It was reported in Fireman-Shoresh *et al.* [86] that sol-gel materials can be tailored for adsorption and separation purposes *via* the molecular imprinting strategy. This group [87] successfully synthesized molecularly imprinted enantio selective polymer films to differentiate enantiomeric pairs following sol-gel route. The designed layer shows selective re-inclusion of that particular compound over the opposite enantiomer, which was not used in imprinting procedures. A model diagram for an enantiomeric sensor is shown in Figure 9. The sensitive and selective discrimination between different enantiomeric pairs is achieved by generating chiral domains in the imprinted polymer. It is courtesy of sol-gel and imprinting techniques that has allowed us the tailoring of final products of required features. In fact, mild reaction conditions and a wide range of metal alkoxide precursors of sol-gel method encourage the development of chiral films. The same group [88] demonstrated the first successful imprinting of an organometallic compound such as ferrocene derivative. In this study, chiral selection properties were measured by cyclic and square voltammetric techniques. These enantio selective imprinted sol-gel layers are highly sensitive and shows excellent signal at 1 nM (0.2 ppb) concentration level. Moreover, significant selectivity pattern is observed when the sensitive layer is exposed to structurally achiral compound of same class.

The contribution of molecularly imprinted sol-gel materials in developing different sensor systems have been investigated by different groups [89,90,91] and numerous applications in biosensors, enzymatic catalysis, electro catalysis and many other fields have been found.

## 5. Conclusions and Future Prospects

Sol-gel technology has the potential to produce highly innovative materials for different sensing applications. The outstanding advantage of this technique is the tailoring of sensitive material according to desired interest. In this article, a significant number of sensor applications are reported, which shows the increasing interest in this field. These include pH sensors, sensors for ionic species, gas phase sensors and some biological sensors. Molecular imprinting plays an important role in designing sol-gel based sensor materials. However, the concept of molecular imprinting in sol-gel science is still in infancy, but is very promising for the development of robust sensor materials. The recent reviews on imprinting strategy in sol-gel matrices shows growing demand in this field for sensor designing. The combination of sol-gel technology with molecular imprinting provides a new synthetic route to improve conventional molecular imprinted polymers and to explore new sensor applications in different fields. The study shows that by modifying the precursors, more stable and strong surfaces can be crafted particularly for high temperature working environments. The use of such sensor systems is significantly important for pollution control and environmental monitoring. Although the critical aspect in designing these sensors for the commercial point of view has not been explained, nevertheless, this emerging field of sensor material is developing and in the future they will be available in the market.
